# Cost-Effectiveness Analysis of Annual vs Biennial Fecal Immunochemical Testing for Colorectal Cancer Screening in Japan: A Microsimulation Analysis

**DOI:** 10.36469/001c.162580

**Published:** 2026-06-26

**Authors:** Taichi Mori, Nobuyuki Shimohata, Rei Goto

**Affiliations:** 1 Graduate School of Health Management Keio University, Kanagawa, Japan; 2 Graduate School of Health Innovation Kanagawa University of Human Services, Kawasaki, Japan; 3 Keio University, Kanagawa, Japan

**Keywords:** fecal immunochemical test, screening interval, microsimulation, cost-effectiveness analysis

## Abstract

**Background:**

The optimal screening interval for fecal immunochemical testing (FIT) in Japan remains uncertain.

**Objective:**

This study aimed to evaluate the cost-effectiveness of biennial FIT screening compared with annual FIT screening to inform future national guidelines.

**Methods:**

We developed a microsimulation model of a cohort of 100 000 average-risk Japanese individuals aged 40 years and older followed over a 60-year horizon from a healthcare payer perspective. Two screening methods were compared: the current annual FIT and biennial FIT. Effectiveness was measured in quality-adjusted life-years (QALYs), and costs were measured in 2024 Japanese yen.

**Results:**

Annual FIT screening was dominant, resulting in lower total costs (¥408 058 vs. ¥451 407) and higher QALYs (26.483 vs 26.440) compared with biennial screening. Probabilistic sensitivity analysis indicated that annual screening was cost-effective in 92.7% of iterations at a WTP threshold of ¥5 million per QALY.

**Conclusions:**

The analysis indicated that annual FIT screening was dominant over biennial screening, because of its lower costs and higher QALY yield. These findings were supported by probabilistic sensitivity analysis. Although annual FIT screening is generally superior to biennial screening in Japan and provides greater health benefits at lower costs, future policies should aim to address endoscopic capacity constraints to ensure program sustainability.

## BACKGROUND

Colorectal cancer (CRC) remains a major public health concern because of its persistently high mortality rate. This has led to the recommendation for population-based screening using fecal immunochemical tests (FIT).^1,2^ Worldwide screening programs differ in target age, screening interval, number of stool samples, and cutoff values. Notably, the screening interval for immunochemical methods strongly influences the total number of follow-up total colonoscopies (TCSs) procedures required and therefore must be considered in the context of available healthcare resources.^3^

Previous studies comparing annual and biennial FIT screening in other settings demonstrated no significant differences in mortality reduction, suggesting the potential for interval optimization without compromising efficacy.^4,5^ Therefore, the Japanese Guidelines for Colorectal Cancer Screening (2024) suggest that biennial FIT screening may be possible, recommending further microsimulation studies within the Japanese context to rigorously examine appropriate screening intervals and resource implications.^3^

Despite evidence that annual and biennial screening do not exhibit a statistically significant difference in mortality reduction,^5^ the exact impact of changing the existing FIT screening interval in Japan on the required number of TCS procedures and the resulting cost-effectiveness remains unclear. This lack of data appears to hinder evidence-based decision-making regarding the optimal allocation of resources for a national screening program.

This study evaluated the cost-effectiveness of biennial FIT screening vs annual FIT screening in Japan, and the potential savings in the required number of TCS procedures associated with the biennial screening. The findings will provide essential evidence to inform the selection of the optimal screening interval for a national CRC screening program.

## METHODS

We developed a microsimulation-based decision model to estimate the long-term clinical and economic impacts of CRC screening in the target population. The model structure draws on empirical evidence aligned with the reporting standards of the CHEERS statement.^6^ Following the Japanese guidelines,^7^ we adopted the healthcare payer perspective. Future costs and health outcomes were discounted at an annual rate of 2%. Comprehensive model inputs, including transition probabilities, costs, and utilities, are detailed in **Supplementary Table S1**. TreeAge Pro 2025 (Build-Id: 25.2.0-v20250709-bd17e8392d) was used for all modeling tasks and simulations.

### Target Population

**Figure 1.**
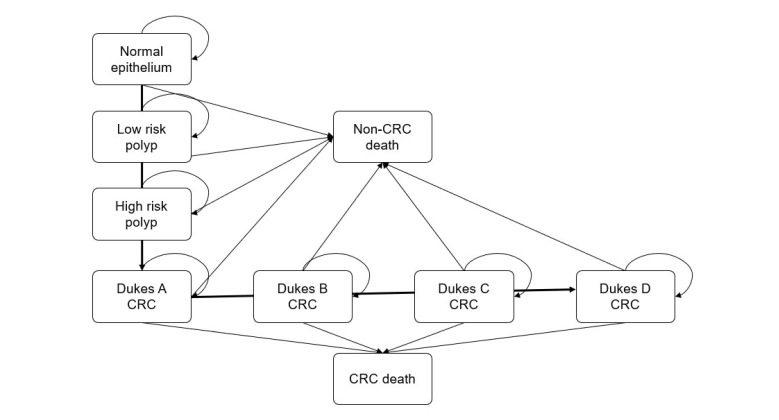
Microsimulation Model for Evaluating CRC Screening and Treatment Cost-Effectiveness This diagram depicts the model framework simulating the natural history of CRC. Grounded in the adenoma-carcinoma sequence, the model encompasses health states ranging from normal epithelium to polyps, as well as the 4 stages of CRC. Arrows represent annual state transitions, covering screening, therapeutic interventions, surveillance, and mortality, allowing for a robust assessment of long-term clinical and economic outcomes. Normal epithelium, disease-free mucosal state; low-risk polyps, adenomas measuring 6-9 mm; high-risk polyps, adenomas ≥10 mm; Dukes A-D CRC, Cancer stages defined by Dukes classification; non-CRC death, mortality from competing causes; CRC death, mortality attributable to CRC.

We simulated a hypothetical cohort of 100 000 average-risk Japanese adults aged 40 years and older, followed over a 60-year time horizon. This time frame was selected to encompass the natural history of CRC, including adenoma formation, progression to malignancy, treatment outcomes, and mortality reduction associated with screening (**Figure 1**).^8^

### Setting and Location

This analysis reflects the current Japanese healthcare system, in which CRC screening is incorporated into the national framework. Screening responsibility is divided as follows: municipalities conduct population-based screening mandated by the Health Promotion Law, while employers and health insurance organizations oversee workplace screening programs.^2^

### Screening

**Figure 2.**
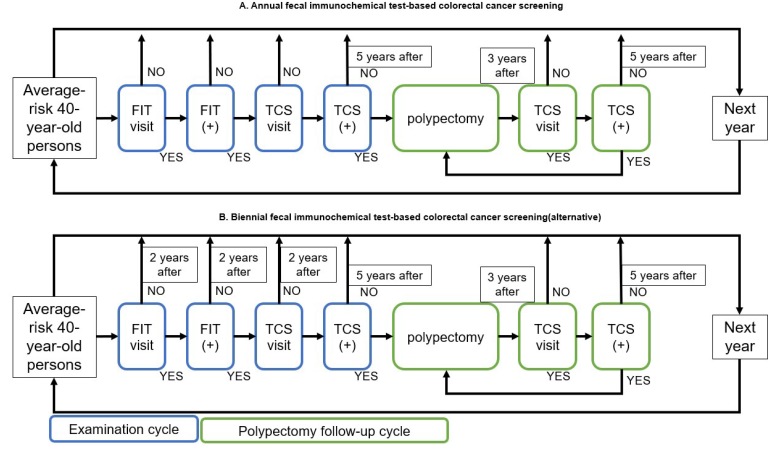
Annual and Biennial FIT-Based Colorectal Cancer Screening Flowchart Abbreviations: FIT, fecal immunochemical test. FIT (+): FIT positive. TCS (+): TCS positive; TCS, total colonoscopy.

Two screenings were compared in this analysis:

1. Annual FIT-based screening (current practice): This screening reflects Japan’s existing national program for individuals aged 40 years and older (**Figure 2A**). The protocol consists of an annual 2-day FIT for asymptomatic adults. A positive result triggers a referral for diagnostic TCS. Post-colonoscopy management was modeled as follows: individuals with normal TCS findings resume screening after a 5-year interval, while those undergoing polypectomy enter a 3-year surveillance interval.^9,10^ Real-world adherence rates were applied at both screening and follow-up steps. Specifically, the base-case participation rate for the initial and subsequent screening rounds was set at 45.95% for both the annual and biennial FIT screenings. This baseline value was derived from the 2022 Comprehensive Survey of Living Conditions by the Ministry of Health, Labour and Welfare, which reflects the current real-world CRC screening uptake in Japan.^11^ Additionally, the follow-up TCS rate after a positive FIT was set at 70.4%, based on the Report on Regional Public Health Services and Health Promotion Services in 2023.^12^ We assumed an equal participation rate across both screenings to isolate and directly compare the intrinsic cost-effectiveness of the screening intervals without the confounding effects of assumed behavioral changes.
2. Biennial FIT-based screening (alternative): Biennial screening is a systematic screening approach (**Figure 2B**) targeting asymptomatic individuals aged 40 years and older and using FIT conducted over 2 consecutive days. The key difference from the existing annual FIT screening program (current practice) is the screening interval, which is extended to once every 2 years. Individuals with positive FIT results were referred for diagnostic TCS, and surveillance followed standard Japanese guidelines, with reentry into the FIT screening arm occurring biennially.

### Disease Progression and Treatment Model

Based on the adenoma-carcinoma sequence (**Figure 1**), the model simulated disease progression through normal mucosa, adenomas (low- and high-risk), and CRC stages (Dukes A-D), including treatment and recovery phases. Dukes staging was adopted for consistency with Japanese Cancer Registry conventions and to maintain model parsimony, following established methodologies in previous colorectal cancer screening studies.^13^ Interventions were size-dependent: cold polypectomy for low-risk polyps (6-9 mm)^14^ and EMR/ESD for high-risk lesions (≥10 mm).^14,15^ Patients with CRC received standard care per Japanese guidelines.^16^ While adenoma progression rates were adopted from the literature^17^ and background mortality from life tables,^18^ calibration was necessary for parameters with insufficient epidemiological evidence. Specifically, disease initiation, stage progression, and symptomatic presentation rates were estimated by calibrating the model to match National Cancer Center statistics.^19^

### Sensitivity and Specificity of FIT and TCS

Diagnostic accuracy parameters (sensitivity and specificity) for both FIT and TCS were obtained from established clinical studies.^20,21^

For TCS, sensitivity was modeled according to lesion size: 92.7% (95% confidence interval [CI], 80.1%-98.5%) for small polyps (6-9 mm) and 97.8% (95% CI, 88.5%-99.9%) for larger polyps (≥10 mm) and all CRC stages.^20^ TCS specificity was 95.8% (95% CI, 92.6%-97.9%).^20^

Regarding FIT, base sensitivities for the single-day method were 24.5% (95% CI, 16.5%-34.0%), 27% (95% CI, 21%-33%), and 71% (95% CI, 56%-83%) for 6-9 mm polyps, polyps ≥10 mm, and CRC, respectively,^20,21^ with a specificity of 95% (95% CI, 94%-96%).^21^ To prevent compounding structural complexity, the model assumed that FIT sensitivity was uniform throughout the colon and rectum, omitting potential variations across specific anatomical locations or detailed histological subtypes. In the standard Japanese 2-day FIT protocol, a screening result is defined as positive if at least 1 of the 2 stool samples tests positive. Because bleeding from colorectal lesions is often intermittent, taking samples over 2 consecutive days increases the overall detection rate. To account for this in our model, we converted the single-day sensitivities to 2-day sensitivities. This conversion assumed that the bleeding events (and subsequent FIT positivity) on consecutive days are statistically independent. Under this assumption of independent events, the probability that at least 1 of the 2 samples tests positive was calculated using the following probabilistic formula:

$$1-(1-\text{Single-Day Sensitivity}{)}^{2}$$

### Health Benefits

Screening effectiveness was quantified as quality-adjusted life-years (QALYs), calculated by weighting the duration spent in each health state by its specific utility value. Utility parameters were obtained from existing literature,^22,23^ where Japanese patient data collected via the EQ-5D-5L instrument were mapped to scores using the Japanese value set.^24,25^

### Costs

Economic analysis included direct healthcare costs and screening costs. All monetary values were denominated in 2024 Japanese yen (¥) and subsequently converted to US dollars ($) by applying the 2024 average exchange rate of ¥151.353 per $1.000.^26^ Unit costs for screening and endoscopic resections were determined based on the national medical fee schedule set by the Ministry of Health, Labour and Welfare. Specifically, polypectomy costs reflect guideline-recommended techniques such as cold polypectomy for low-risk adenomas and EMR/ESD for high-risk lesions. CRC treatment costs were obtained from the published literature.^27^ To standardize the values, historical cost data were inflation-adjusted to 2024 levels using the Ministry of Health, Labour and Welfare’s medical care reimbursement revision indices.

### Cost-Effectiveness, Sensitivity, and Scenario Analyses

Economic efficiency was evaluated using the incremental cost-effectiveness ratio (ICER), defined as the additional cost per QALY. Screening was considered cost-effective when the ICER was below the willingness-to-pay (WTP) threshold of ¥5 million ($33 035) per QALY.^7^ Model robustness was assessed using deterministic sensitivity analysis (DSA) and probabilistic sensitivity analysis (PSA). In the PSA (1000 Monte Carlo iterations), parameter distributions were based on 95% CIs; when the 95% CI or standard error (SE) was unavailable, the 95% CI was calculated using 20% of the mean as the SE. All parameters were assumed to be mutually independent, and no correlations between parameters were considered in the PSA. Furthermore, to ensure the stability of the simulation outcomes and minimize Monte Carlo sampling error, we performed PSA convergence diagnostics. We plotted the cumulative mean of the incremental net monetary benefit alongside its 95% confidence intervals across the iterations. In the DSA, we varied each parameter across its 95% CI. When only the point estimate was available, the SE was assumed to be 20% of the mean, and the range of variation was defined as the point estimate ± 1.96 × SE.

We expanded our analysis to investigate the impact of different screening policies and adherence levels. We compared 4 alternative FIT screening protocols, varying by starting age and interval: annual screening starting at ages 45 and 50 years, and biennial screening starting at ages 45 and 50 years. Additionally, we simulated participation rates ranging from 10% to 100% in 10% increments for both annual and biennial FIT-based screening.

### Calibration

Model calibration was performed to establish internal validity and estimate unobservable parameters, including the initiation rate from normal epithelium to low-risk polyps, stage progression rates, and stage-specific symptomatic presentation rates (Dukes A-D). The calibration targeted the cumulative incidence and mortality risks of CRC expected for a cohort followed from age 40 to 85, expressed as probabilities derived from the 2020 Japanese Cancer Registry and Vital Statistics.^28,29^ We utilized the Bound Optimization BY Quadratic Approximation algorithm to minimize the goodness-of-fit statistic. Detailed descriptions of the targets, optimization outputs, and validation metrics are presented in **Supplementary Table S2**.

### NDB Open Data

To estimate the number of colonoscopies performed in Japan, we utilized the NDB Open Data (National Database of Health Insurance Claims and Specific Health Checkups of Japan) provided by the Ministry of Health, Labour and Welfare.^30^ We aggregated the annual counts of inpatient and outpatient colonoscopy claims (medical billing code 160094710) for fiscal years 2018-2022.

### Use of Artificial Intelligence

During the preparation of this work, the authors used Google Gemini to assist with language editing and to improve the readability of the manuscript. After using this tool, the authors reviewed and edited the content as needed and take full responsibility for the final content of the publication.

## RESULTS

### Base-Case Analysis

In the base-case analysis, annual FIT screening dominated biennial FIT screening (**Table 1**). Total cost per individual was ¥408 058 (approx. $2696) for annual FIT vs ¥451 407 ($2982) for biennial FIT, corresponding to an incremental cost increase of ¥43 349 ($286) with biennial FIT-based screening. In terms of health outcomes, annual FIT yielded 26.483 QALYs vs 26.440 QALYs with biennial FIT, indicating an incremental QALY loss of 0.043 for the biennial screening. Accordingly, biennial FIT screening was dominated by annual FIT screening.

**Table 1.** Base-Case Cost-Effectiveness Results of Annual vs Biennial FIT-Based Screening

**Base-Case Analysis**	**Costs, ¥ ($)**	**Incremental Costs, ¥ ($)**	**QALYs**	**Incremental QALYs**	**ICER, ¥/QALY ($/QALY)**
Annual FIT-based screening^a^	408 058 (2696)		26.48318394		
Biennial FIT-based screening^b^	451 407 (2982)	43 349 (286)	26.44046574	-0.0427182	Dominated^c^

Abbreviations: FIT: fecal immunochemical test; ICER: incremental cost-effectiveness ratio; QALY: quality-adjusted life year.

^a^Refers to current Japanese practice (annual screening for individuals aged ≥40 years).

^b^Represents screening performed every 2 years.

^c^Indicates that the alternative screening (biennial FIT) is more costly and less effective than the current practice (annual FIT).

Costs represent total cumulative costs per person. Currency was converted at a rate of $1 = ¥151.353. Incremental values denote the difference between the biennial and annual strategies.

### Sensitivity Analyses

**Figure 3.**
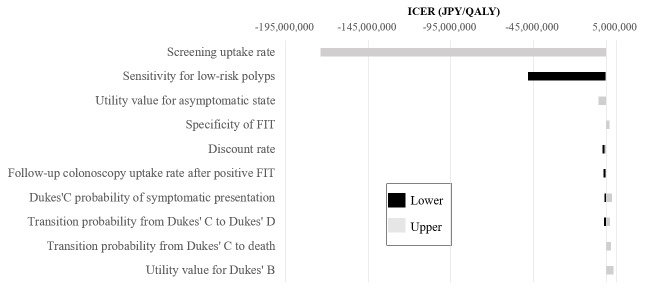
Tornado Diagram of One-Way Sensitivity Analysis in Base Case Abbreviations: FIT, fecal immunochemical test; TCS, total colonoscopy.

**Figure 4.**
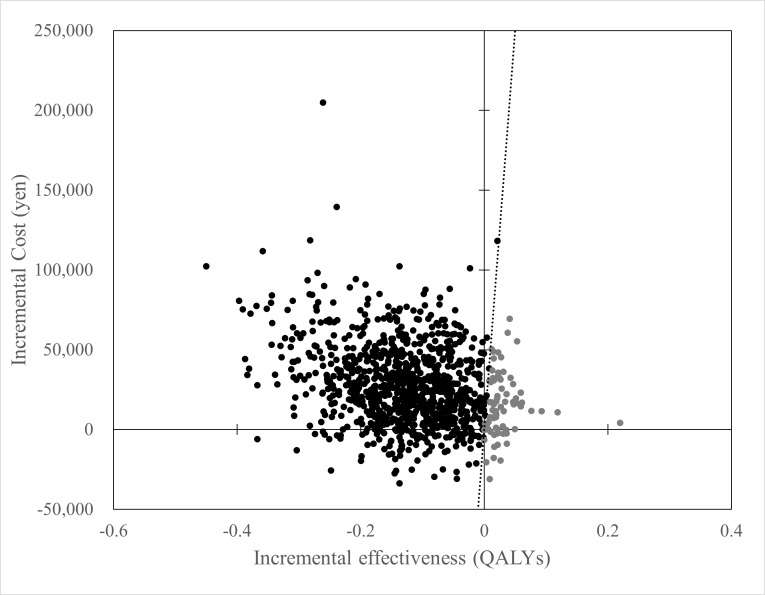
Probabilistic Sensitivity Analysis (Scatterplot) Abbreviations: QALY, quality-adjusted life-years; PSA, probabilistic sensitivity analysis; WTP, willingness to pay.

In the DSA, cost-effectiveness results were most sensitive to the screening uptake rate, followed by FIT sensitivity for low-risk polyps, utility value for asymptomatic state, and specificity of FIT (**Figure 3**). PSA was conducted with 1000 iterations. At a WTP threshold of ¥5 million per QALY, annual FIT-based screening was cost-effective in 92.7% of iterations (**Figure 4**). Convergence diagnostics demonstrated that the cumulative mean incremental net monetary benefit stabilized and its 95% CI remained consistently below the zero threshold, confirming that 1000 iterations were sufficient to achieve robust model convergence (**Supplementary Figure S1**).

The scatterplot illustrates the results of 1000 Monte Carlo simulation iterations on the incremental cost-effectiveness plane. The vertical axis represents the incremental costs (in ¥), and the horizontal axis represents the incremental effectiveness (in QALYs). The black dots represent iterations in which the biennial screening was dominated, whereas the gray dots represent iterations in which the biennial screening was dominant or cost-effective (WTP threshold of ¥5 million per QALY). The diagonal dotted line indicates the WTP threshold of ¥5 million per QALY gained.

### Scenario Analyses

**Figure 5.**
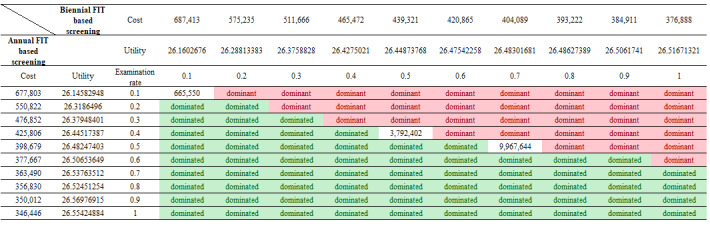
Scenario Analysis of Cost-Effectiveness Across Varying Participation Rates Abbreviation: FIT, fecal immunochemical test. Green (dominated): Biennial FIT-based screening is dominated by annual FIT-based screening (annual is cheaper and more effective). Red (dominant): Biennial FIT-based screening dominates annual FIT-based screening (ie, is cheaper and more effective). Numerical values (white cells): Incremental cost-effectiveness ratio (ICER, ¥/QALY) of biennial relative to annual FIT-based screening when the biennial screening is more effective but more costly. Hyphens indicate scenarios in which the alternative is cost-saving but less effective. The outcomes were assessed against a WTP threshold of ¥5 million/QALY ($33 035/QALY).

We conducted a scenario analysis to assess how participation-rate variation influenced the economic outcomes of annual (current practice) and biennial FIT-based screening (**Figure 5**). While the results generally favored the current practice, the dominance depended on adherence levels. When participation rates were equivalent across screenings, annual FIT-based screening dominated biennial FIT-based screening in most scenarios, except at the lowest participation rate (10%), where the biennial screening was more effective and resulted in an ICER of ¥665 550/QALY. Furthermore, the annual FIT must maintain a high participation rate (≥70%) to remain the dominant screening regardless of the participation rate for the biennial alternative. Conversely, if the current annual participation rate is moderate (eg, 50%) and adopting a biennial interval significantly improves participation (eg, to ≥80%), the biennial screening becomes dominant.

We evaluated the impact of delaying screening initiation to 45 and 50 years for both annual and biennial FIT-based screening. In all cases, delayed initiation reduced cost-effectiveness relative to the base case (**Supplementary Table S3**). For annual FIT-based screening, delaying the start age to 45 years increased total cost to ¥408 555 and reduced effectiveness to 26.479 QALYs; a further delay to age 50 increased cost to ¥425 864 and lowered effectiveness to 26.442 QALYs. Biennial FIT-based screening showed a similar pattern of higher costs, while its QALYs remained nearly equivalent or slightly decreased compared with the baseline. Consequently, all delayed-initiation scenarios (45 or 50 years) were dominated by annual FIT-based screening starting at the age of 40.

### TCS Capacity and Trend

The annual volume of total colonoscopies peaked in 2019 and subsequently declined overall. Although a temporary increase occurred in 2021, the number of procedures performed by 2022 remained below the 2019 level (**Supplementary Figure S2)**. In the base case (starting age, 40 years), the number of TCS procedures was 201,836 under annual screening and 168 082 under biennial screening. When the starting age was shifted to 45 years, the corresponding numbers were 193 136 and 163 146 for the annual and biennial screenings, respectively. When screening initiation was delayed to 50 years, TCS requirements decreased to 178 895 and 152 316 for annual and biennial screening, respectively (**Supplementary Table S3**).

### Validity and Calibration

To verify the predictive accuracy of the model, we compared its output with actual incidence and mortality figures sourced from the Cancer Registry and Statistics.^28,29^ The model-predicted cumulative incidence and mortality risks closely matched the observed registry targets, demonstrating relative errors of -0.6% and +5.2%, respectively, and a minimized goodness-of-fit statistic of 2.27 × 10^-6^ (**Supplementary Table S2**). As depicted in **Supplementary Figure S3** (y-axis: cases or deaths per 10 000), the simulated trends were generally consistent with the external registry data.

## DISCUSSION

This study evaluated the cost-effectiveness of different FIT screening intervals in Japan. The analysis indicates that annual FIT screening dominated biennial screening, because it incurs lower costs and yields higher QALYs. PSA supported the robustness of these findings. DSA demonstrated that screening uptake rate and FIT sensitivity substantially influenced the results. Annual FIT-based screening requires a higher number of follow-up colonoscopies compared with biennial screening.

To our knowledge, this study is the first to evaluate the cost-effectiveness of different FIT screening intervals in Japan.^3^ The 2024 Japanese guidelines suggested biennial FIT as a potential option. However, they required rigorous microsimulation verification using domestic data. Our research directly addresses this critical gap.^3^ We demonstrated the economic dominance of annual screening from a healthcare payer perspective. These results provide vital evidence for future national policy decisions.

Furthermore, our findings align with existing international evidence supporting annual FIT. A Chinese Markov model study by Ren et al found that annual FIT dominated biennial screening, which is consistent with our simulation results.^31^ Similarly, a Slovakian MISCAN-Colon analysis by Babela et al showed annual FIT was highly cost-effective, reducing mortality at an increased total cost compared with biennial screening.^32^ Furthermore, a European systematic review concluded that annual FIT is the most cost-effective screening under unconstrained resource conditions.^33^ Overall, our Japanese simulation results seem to agree with these global findings.

Participation rates heavily influence the cost-effectiveness of screening. Although our base-case analysis assumed equal participation for both intervals to isolate intrinsic cost-effectiveness without confounding behavioral changes, a longer biennial interval could potentially increase screening participation. For example, European biennial programs achieve approximately 50% participation.^34^ However, our scenario analysis demonstrated the robustness of the annual screening. Annual FIT remains economically dominant even if biennial participation increases to 60%. Biennial screening requires a massive participation increase to over 80% to become dominant. Therefore, annual FIT provides superior health benefits at lower costs in Japan unless adherence jumps drastically.

Healthcare resource constraints highlight a critical trade-off in determining the optimal screening policy. We evaluated different starting ages and screening intervals in our scenario analysis. Annual FIT starting at age 40 was the most cost-effective screening. Annual screenings starting at ages 45 and 50 followed in economic efficiency. Annual screening completely dominated all biennial screenings. However, these highly cost-effective annual screenings require a high volume of follow-up colonoscopies. Delaying the starting age or extending the interval reduced this colonoscopy burden.

Resource constraints often dictate national screening policies. Several European countries choose biennial FIT to manage limited endoscopic capacity.^33^ Japanese NDB Open Data shows a declining trend in annual colonoscopy volumes (**Supplementary Figure S2**). This trend might indicate a decrease in future endoscopic resources. Assessing the current and future TCS capacity in Japan is a critical task.^9^ If capacity becomes constrained, biennial screening or a later starting age are realistic options. These adjustments could help maintain the sustainability of the national screening program.

This study has several limitations. First, we focused on the adenoma-carcinoma sequence. Our model omits alternative pathways, such as de novo carcinogenesis and the serrated pathway. These structural simplifications might influence the predicted outcomes relative to known colorectal cancer biology.

Second, we assumed equal participation rates for both screenings in the base case. In reality, adherence may vary between annual and biennial intervals. We also did not model individual-level behaviors. For instance, people who attend one round are often more likely to return for future tests. We excluded these tendencies to maintain model parsimony.

Third, our findings may lack generalizability across all screening contexts in Japan. The model does not differentiate between workplace and community-based programs. We also overlooked potential adherence gaps among different socioeconomic groups or geographic regions. Such variations could impact the equitable distribution of health benefits. These factors might affect real-world cost-effectiveness outcomes.

Fourth, we did not explicitly account for total colonoscopy capacity. However, resource limits could affect screening feasibility. Specific capacity settings might alter the relative dominance of annual and biennial screenings. Future research should integrate Japanese-specific resource constraints to ensure policy sustainability.

Fifth, our model relied on size-based FIT sensitivity and assumed uniform diagnostic performance across all colorectal segments. In reality, FIT sensitivity varies by anatomical location and histological pathways. Omission of these heterogeneities to maintain model parsimony may oversimplify real-world dynamics, potentially influencing cost-effectiveness outcomes. Future studies should incorporate these granular variations as robust domestic data become available.

Finally, some parameters were derived from the literature or estimated through calibration. This approach introduces potential uncertainty in the precision of our estimates. While our base-case results favor annual screening, these limitations warrant a cautious interpretation. Future studies with more granular data may further refine these conclusions.

## CONCLUSIONS

The current practice of annual FIT screening was more cost-effective than biennial screening; however, the availability of endoscopic resources significantly affects this conclusion. Future policy planning should prioritize addressing resource constraints to ensure program sustainability.

### Conflicts of Interest

The authors declare no conflicts of interest.

## Supplementary Material

Online Supplementary Material
