# Supplementary material for: Cost-Effectiveness Analysis of Annual vs Biennial Fecal Immunochemical Testing for Colorectal Cancer Screening in Japan: A Microsimulation Analysis
Source: J Health Econ Outcomes Res. 2026 Jun 26;13(1):272–9. doi: 10.36469/001c.162580 (PMC13310510; doi:10.36469/001c.162580)
Supplement: Online Supplementary Material [file jheor_2026_13_1_162580_351302.pdf]

Journal of Health Economics and Outcomes Research

Online Supplementary Material

Cost-Effectiveness Analysis of Annual Versus Biennial Fecal Immunochemical Testing for Colorectal Cancer Screening in Japan: A Microsimulation Analysis. *JHEOR*. 2026;13(1):272-279. doi:10.36469/jheor.2026.162580

Figure S1: Convergence Plot for the Probabilistic Sensitivity Analysis **1**

Figure S2: Inpatient and Outpatient Colonoscopies Performed Annually in Japan **2**

Figure S3: Validation of the Microsimulation Model Against Japanese Registry Data **3**

Table S1: Input Parameters Used in the Microsimulation Model: Annual Transition Probabilities, Diagnostic Performance, Healthcare Costs, and Health State Utility Values **4**

Table S2: Calibration Targets, Model-Predicted Outputs, and Goodness-of-Fit **11**

Table S3: Scenario Analysis: Impact of Varying Screening Initiation Ages on Cost-Effectiveness and Resource Requirements **12**

References **13**

This supplementary material has been provided by the authors to give readers additional information about their work.

This is an open-access article distributed under the terms of the Creative Commons Attribution 4.0 International License (CCBY-4.0). View this license's legal deed at http://creativecommons.org/licenses/by/4.0 and legal code at http://creativecommons.org/licenses/by/4.0/legalcode for more information.

Probabilistic Sensitivity Analysis Convergence Diagnostics

To verify the adequacy of the 1,000 iterations used in the probabilistic sensitivity analysis (PSA), we assessed model convergence using the incremental net monetary benefit (INMB) framework. A convergence plot (**Figure S1**) was generated to track the cumulative mean INMB and its standard error-derived 95% confidence intervals at a willingness-to-pay threshold of ¥5 million per quality-adjusted life-year (QALY). The plot shows that the 95% confidence intervals narrowed progressively and did not cross the zero threshold, indicating that the decision uncertainty due to Monte Carlo sampling error was effectively minimized.

Figure S1. Convergence Plot for the Probabilistic Sensitivity Analysis

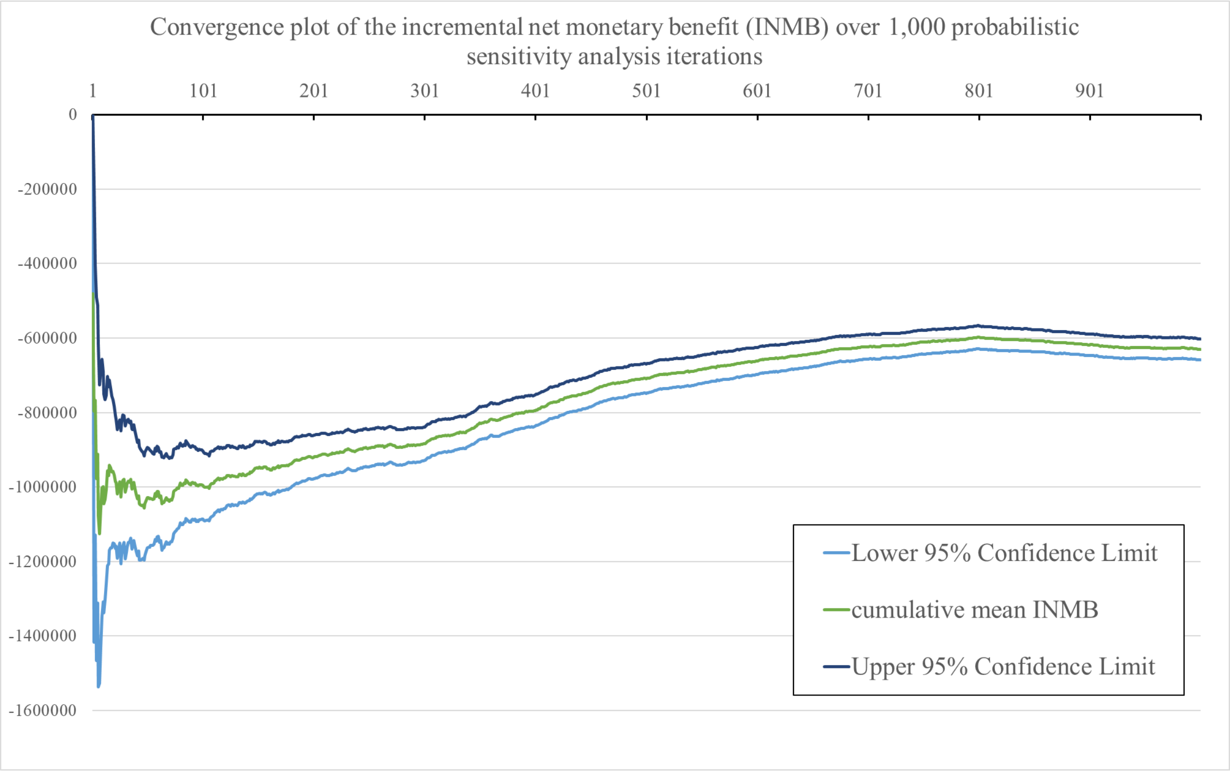

The plot illustrates the cumulative mean incremental net monetary benefit (INMB) (green line) and its 95% confidence intervals (blue and dark blue lines) over 1,000 Monte Carlo iterations comparing biennial vs annual FIT-based screening. The stability of the lines confirms robust model convergence.

Figure S2. Inpatient and Outpatient Colonoscopies Performed Annually in Japan

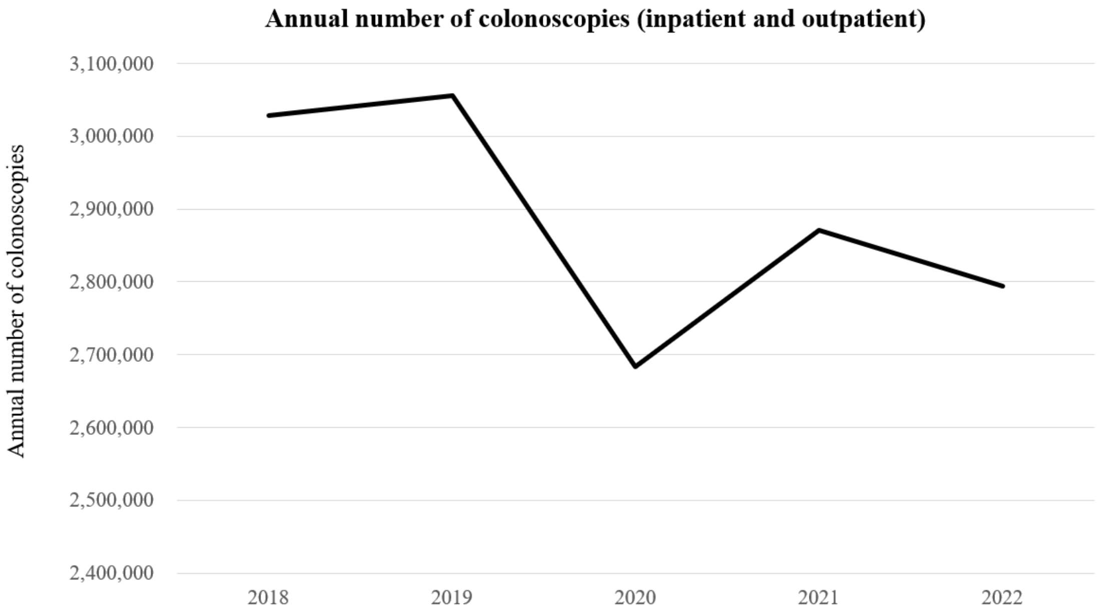

Abbreviation**:** TCS, total colonoscopy.

The line illustrates the total number of colonoscopies (TCS) performed annually in Japan, combining both inpatient and outpatient settings. The vertical axis represents the annual volume of procedures. A notable decline is observed in 2020, followed by a fluctuation in subsequent years.

Figure S3. Validation of the Microsimulation Model Against Japanese Registry Data

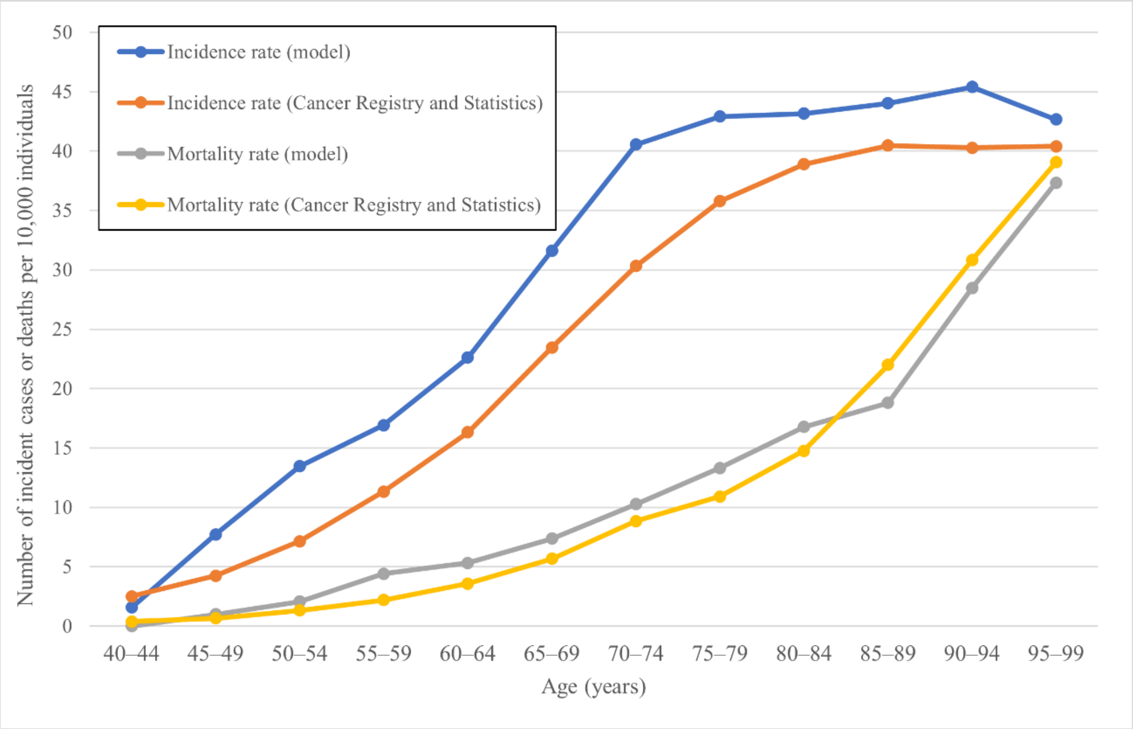

This figure plots age-specific colorectal cancer incidence and mortality rates to assess model performance. The x-axis represents 5-year age bands, and the y-axis indicates the number of cases or deaths per 10,000 individuals. Model-estimated incidence (blue dot line) and mortality (gray dot line) are compared with observed data from the Cancer Registry and Statistics of Japan (incidence, orange line; mortality, yellow line).

Table S1. Input Parameters Used in the Microsimulation Model: Annual Transition Probabilities, Diagnostic Performance, Healthcare Costs, and Health State Utility Values

	Base-Case Value	Sensitivity Analysis Range	Distribution	Reference
**Transition probabilities (per year)**				
Probability from normal epithelium to low-risk polyp per year	0.089105	0.05418-0.12403	Beta (SE: 0.01782)	Calibration [1-3]
Probability from low-risk polyp to high-risk polyp per year	0.0415	0.025232-0.057768	Beta (SE: 0.0083)	Assumption [4]
Probability from high-risk polyp to Dukes A CRC per year	0.0255 (40 - 59)<br>0.029 (60 - 64)<br>0.038 (65 - 69)<br>0.0505 (70 - 74)<br>0.054 (75-)	0.015504-0.035496,<br>0.017632-0.040368,<br>0.023104-0.052896,<br>0.030704-0.070296,<br>0.032832-0.075168	Beta (SE: 0.0051),<br>Beta (SE: 0.0058),<br>Beta (SE: 0.0076),<br>Beta (SE: 0.0101),<br>Beta (SE: 0.0108)	[4]
Probability from Dukes A to B per year	0.671669	0.40838-0.93496	Beta (SE: 0.13433)	Calibration [1-3]
Probability from Dukes B to C per year	0.715852	0.43524-0.99647	Beta (SE: 0.14317)	Calibration [1-3]
Probability from Dukes C to D per year	0.854471	0.51952-1	Beta (SE: 0.17089)	Calibration [1-3]
Probability from Dukes A to death per year	0.0177	0.0107616-0.0246384	Beta (SE: 0.00354)	[5]
Probability from Dukes B to death per year	0.0322	0.0195776-0.0448224	Beta (SE: 0.00644)	[5]
Probability from Dukes C to death per year	0.0712	0.0432896-0.0991104	Beta (SE: 0.01424)	[5]
Probability from Dukes D to death per year	0.2906	0.1766848-0.4045152	Beta (SE: 0.05812)	[5]
Probability of recurrence after treatment of colorectal cancer (Dukes A)	0.0196	0.0119168-0.0272832	Beta (SE: 0.00392)	[5]
Probability of recurrence after treatment of colorectal cancer (Dukes B)	0.035	0.02128-0.04872	Beta (SE: 0.007)	[5]
Probability of recurrence after treatment of colorectal cancer (Dukes C)	0.0786	0.0477888-0.1094112	Beta (SE: 0.01572)	[5]
Probability of recurrence after treatment of colorectal cancer (Dukes D)	0.120318	0.07315-0.16748	Beta (SE: 0.02406	Calibration [1-3]
Probability of symptomatic	0.145852	0.08868-0.20303	Beta (SE: 0.02917)	Calibration, [1-3]

	Base-Case Value	Sensitivity Analysis Range	Distribution	Reference
presentation of CRC: Dukes A				
Probability of symptomatic presentation of CRC: Dukes B	0.250575	0.15235-0.3488	Beta (SE: 0.05011)	Calibration [1-3]
Probability of symptomatic presentation of CRC: Dukes C	0.409228	0.24881-0.56965	Beta (SE: 0.08185)	Calibration [1-3]
Probability of symptomatic presentation of CRC: Dukes D	0.906323	0.55104-1	Beta (SE: 0.18126)	Calibration [1-3]
**Natural mortality rate**				
40	0.000731782	0.00044-0.00102	Beta (SE: 0.00015)	Assumption [3,6]
41	0.000798335	0.00049-0.00111	Beta (SE: 0.00016)	
42	0.000875212	0.00053-0.00122	Beta (SE: 0.00018)	
43	0.0009472	0.00058-0.00132	Beta (SE: 0.00019)	
44	0.001024489	0.00062-0.00143	Beta (SE: 0.0002)	
45	0.001090701	0.00066-0.00152	Beta (SE: 0.00022)	
46	0.001199118	0.00073-0.00167	Beta (SE: 0.00024)	
47	0.001328348	0.00081-0.00185	Beta (SE: 0.00027)	
48	0.001483678	0.0009-0.00207	Beta (SE: 0.0003)	
49	0.00165505	0.00101-0.0023	Beta (SE: 0.00033)	
50	0.00176044	0.00107-0.00245	Beta (SE: 0.00035)	
51	0.001954174	0.00119-0.00272	Beta (SE: 0.00039)	
52	0.002154239	0.00131-0.003	Beta (SE: 0.00043)	
53	0.002381416	0.00145-0.00331	Beta (SE: 0.00048)	
54	0.002610258	0.00159-0.00363	Beta (SE: 0.00052)	
55	0.002790003	0.0017-0.00388	Beta (SE: 0.00056)	
56	0.003069721	0.00187-0.00427	Beta (SE: 0.00061)	
57	0.003378229	0.00205-0.0047	Beta (SE: 0.00068)	
58	0.003700391	0.00225-0.00515	Beta (SE: 0.00074)	
59	0.004052552	0.00246-0.00564	Beta (SE: 0.00081)	
60	0.004297313	0.00261-0.00598	Beta (SE: 0.00086)	
61	0.004701265	0.00286-0.00654	Beta (SE: 0.00094)	
62	0.005180527	0.00315-0.00721	Beta (SE: 0.00104)	
63	0.005737122	0.00349-0.00799	Beta (SE: 0.00115)	
64	0.006368242	0.00387-0.00886	Beta (SE: 0.00127)	
65	0.006840957	0.00416-0.00952	Beta (SE: 0.00137)	
66	0.007564242	0.0046-0.01053	Beta (SE: 0.00151)	
67	0.008315322	0.00506-0.01157	Beta (SE: 0.00166)	
68	0.00917469	0.00558-0.01277	Beta (SE: 0.00183)	
69	0.010181581	0.00619-0.01417	Beta (SE: 0.00204)	
70	0.011026636	0.0067-0.01535	Beta (SE: 0.00221)	
71	0.012343935	0.00751-0.01718	Beta (SE: 0.00247)	
72	0.013786678	0.00838-0.01919	Beta (SE: 0.00276)	
73	0.01540183	0.00936-0.02144	Beta (SE: 0.00308)	
74	0.017205503	0.01046-0.02395	Beta (SE: 0.00344)	

	Base-Case Value	Sensitivity Analysis Range	Distribution	Reference
75	0.018852618	0.01146-0.02624	Beta (SE: 0.00377)	
76	0.020827503	0.01266-0.02899	Beta (SE: 0.00417)	
77	0.023152982	0.01408-0.03223	Beta (SE: 0.00463)	
78	0.025969279	0.01579-0.03615	Beta (SE: 0.00519)	
79	0.029273423	0.0178-0.04075	Beta (SE: 0.00585)	
80	0.032670376	0.01986-0.04548	Beta (SE: 0.00653)	
81	0.036824741	0.02239-0.05126	Beta (SE: 0.00736)	
82	0.041250474	0.02508-0.05742	Beta (SE: 0.00825)	
83	0.046445015	0.02824-0.06465	Beta (SE: 0.00929)	
84	0.052654483	0.03201-0.0733	Beta (SE: 0.01053)	
85	0.059192306	0.03599-0.0824	Beta (SE: 0.01184)	
86	0.067448035	0.04101-0.09389	Beta (SE: 0.01349)	
87	0.076664345	0.04661-0.10672	Beta (SE: 0.01533)	
88	0.087113784	0.05297-0.12126	Beta (SE: 0.01742)	
89	0.098962355	0.06017-0.13776	Beta (SE: 0.01979)	
90	0.111308005	0.06768-0.15494	Beta (SE: 0.02226)	
91	0.124283964	0.07556-0.173	Beta (SE: 0.02486)	
92	0.139363412	0.08473-0.19399	Beta (SE: 0.02787)	
93	0.155234958	0.09438-0.21609	Beta (SE: 0.03105)	
94	0.172831371	0.10508-0.24058	Beta (SE: 0.03457)	
95	0.196299984	0.11935-0.27325	Beta (SE: 0.03926)	
96	0.220704129	0.13419-0.30722	Beta (SE: 0.04414)	
97	0.247283355	0.15035-0.34422	Beta (SE: 0.04946)	
98	0.276140595	0.16789-0.38439	Beta (SE: 0.05523)	
99	0.307174345	0.18676-0.42759	Beta (SE: 0.06143)	
100	0.340415137	0.20697-0.47386	Beta (SE: 0.06808)	
**Costs, ¥ ($)**				
FIT cost	1,080 (7)	657-1,503	Gamma (SE: 216)	Assumption,[7]
TCS cost	32,320 (214)	19,651-44,989	Gamma (SE: 6,464)	Assumption,[7]
Cost of endoscopic resection of low-risk polyp	74,600 (493)	45,357-103,843	Gamma (SE: 14,920)	Assumption [7]
Cost of endoscopic resection of high-risk polyp	458,693 (3,031)	278,885-638,501	Gamma (SE: 91,739)	[8]
Annual cost management for patients with Dukes A stage (1st year)	1,808,882 (11,951)	1,099,800-2,517,964	Gamma (SE: 361,776)	[8]
Annual cost management for patients with Dukes B stage (1st year)	2,082,459 (13,759)	1,266,135-2,898,783	Gamma (SE: 416,492)	[8]
Annual cost management for patients with Dukes C stage (1st year)	2,670,545 (17,644)	1,623,691-3,717,399	Gamma (SE: 534,109)	[8]
Annual cost management for	3,217,998 (21,262)	1,956,543-4,479,453	Gamma (SE: 643,600)	[8]

	Base-Case Value	Sensitivity Analysis Range	Distribution	Reference
patients with Dukes D stage (1st year)				
Annual management cost for patients with Dukes A stage (years 2+)	34,439 (228)	20,939-47,939	Gamma (SE: 6,888)	[2]
Annual management cost for patients with Dukes B stage (years 2+)	34,439 (228)	20,939-47,939	Gamma (SE: 6,888)	[2]
Annual management cost for patients with Dukes C stage (years 2+)	43,543 (288)	26,474-60,612	Gamma (SE: 8,709)	[2]
Annual management cost for patients with Dukes D stage (years 2+)	2,464,080 (16,280)	1,498,161-3,429,999	Gamma (SE: 492,816)	[2]
Cost of perforation after EMR	208,530 (1,378)	126,786-290,274	Gamma (SE: 41,706)	Assumption [7]
**Probability**				
Examination rate for FIT	0.4595	0.279376-0.639624	Beta (SE: 0.0919)	[9]
Examination rate for TCS after FIT	0.704	0.428032-0.979968	Beta (SE: 0.1408)	[9,10]
FIT sensitivity for low−risk polyps	0.245	0.165-0.34	Beta (SE: 0.044643)	[11]
TCS sensitivity for low−risk polyps	0.927	0.801-0.985	Beta (SE: 0.046939)	[11]
FIT sensitivity for high−risk polyps	0.27	0.21-0.33	Beta (SE: 0.030612)	[12]
TCS sensitivity for high-risk polyps	0.978	0.885-0.999	Beta (SE: 0.029082)	[11]
TCS sensitivity for CRC (Dukes A-D)	0.978	0.885-0.999	Beta (SE: 0.029082)	[11]
FIT sensitivity for CRC (Dukes A-D)	0.71	0.56-0.83	Beta (SE: 0.068878)	[12]
TCS specificity for colorectal polyps and CRC	0.958	0.926-0.979	Beta (SE: 0.0135)	[11]
FIT specificity for colorectal polyps and CRC	0.95	0.94-0.96	Beta (SE: 0.0051)	[12]
Probability of adverse events following TCS	0.00011	0.00006688-0.00015312	Beta (SE: 0.000022)	[13]
Probability of death following perforation	0.0365	0.022192-0.050808	Beta (SE: 0.0073)	[13]
Probability of perforation after TCS	0.0069	0.0041952-0.0096048	Beta (SE: 0.00138)	[14]

	Base-Case Value	Sensitivity Analysis Range	Distribution	Reference
with endoscopic polyp resection				
**Utility**				
Healthy Japanese population	0.891	0.757-1	Beta (SE: 0.13)	[15,16]
Normal epithelium	0.891	0.757-1	Beta (SE: 0.13)	[15,16]
Low risk polyp	0.891	0.757-1	Beta (SE: 0.13)	[15,16]
High risk polyp	0.891	0.757-1	Beta (SE: 0.13)	[15,16]
Polypectomy	0.891	0.757-1	Beta (SE: 0.13)	[15,16]
Dukes A colorectal cancer	0.879	0.534432-1	Beta (SE: 0.1758)	[17,18]
Dukes B colorectal cancer	0.879	0.534432-1	Beta (SE: 0.1758)	[17,18]
Dukes C colorectal cancer	0.867	0.527136-1	Beta (SE: 0.1734)	[17,18]
Dukes D colorectal cancer	0.867	0.527136-1	Beta (SE: 0.1734)	[17,18]
Dukes A - Follow-up (Years 2-5)	0.879	0.534432-1	Beta (SE: 0.1758)	[17,18]
Dukes B - Follow-up (Years 2-5)	0.879	0.534432-1	Beta (SE: 0.1758)	[17,18]
Dukes C - Follow-up (Years 2-5)	0.867	0.527136-1	Beta (SE: 0.1734)	[17,18]
Dukes D - Follow-up (Years 2-5)	0.867	0.527136-1	Beta (SE: 0.1734)	[17,18]
Post-treatment states	0.891	0.757-1	Beta (SE: 0.13)	[15,16]
Non-CRC death	0			Assumption
CRC death	0			Assumption

Abbreviations: CRC, colorectal cancer; EMR, endoscopic mucosal resection; ESD, endoscopic submucosal dissection; FIT, fecal immunochemical test; QALY, quality-adjusted life-years; TCS, total colonoscopy.

Clinical Definitions

Polyp classification: Low-risk polyps are defined as adenomas 6-9 mm in size; high-risk polyps refer to advanced adenomas ≥ 10 mm.

CRC staging: Dukes A, B, C, and D represent stages of colorectal cancer according to the Dukes classification system. For analysis, these are grouped as Dukes A/B and Dukes C/D.

Calculations and Cost Adjustments:

FIT sensitivity: Sensitivity for the 2-day FIT protocol was calculated as: $1 - (1 - \text{single-day sensitivity})^2$.

Currency conversion: Japanese yen values were converted to US dollars using the 2024 average exchange rate (US $ = ¥151.353).

Cost adjustment: Historical cost data were adjusted to 2024 values based on the official medical care reimbursement revision rates from the Ministry of Health, Labour and Welfare.

Outcomes

QALYs represent an aggregated measure of time spent in each health state, weighted by the health utility value of that state.

Table S2. Calibration Targets, Model-Predicted Outputs, and Goodness-of-Fit

Calibration Target (Age 40-85)	Observed Target Value (Probability)[a]	Model-Predicted Output	Relative Error (%)
Cumulative CRC incidence risk	0.09269	0.0921	-0.64
Cumulative CRC mortality risk	0.0267	0.02809	+5.21

Abbreviation: CRC, colorectal cancer.

[a] Observed target values represent the expected per-person probability derived from 100,000 population-based estimates in the 2020 Japanese Cancer Registry and Vital Statistics. The final optimized goodness-of-fit (sum of squared errors) using the Bound Optimization BY Quadratic Approximation (BOBYQA) algorithm was 2.272×10^{-6}, indicating an excellent model fit.

Calibration Methods and Validation

To estimate natural history parameters lacking direct epidemiological data in Japan, we performed model calibration using a formal goodness-of-fit optimization approach. The primary calibration targets were the cumulative CRC incidence risk (0.09269) and cumulative CRC mortality risk (0.02670) for individuals aged 40 to 85 years, converted to expected probabilities per person based on 2020 national statistics. We employed the Bound Optimization BY Quadratic Approximation (BOBYQA) algorithm to automatically search for the optimal parameter set by minimizing the sum of squared errors between the observed targets and model-predicted outputs. The nine unobservable variables estimated through this process were:

- Annual probability of transition from normal epithelium to low-risk polyp
- Annual probability of progression from Dukes A to Dukes B CRC
- Annual probability of progression from Dukes B to Dukes C CRC
- Annual probability of progression from Dukes C to Dukes D CRC
- Annual probability of symptomatic presentation for Dukes A CRC
- Annual probability of symptomatic presentation for Dukes B CRC
- Annual probability of symptomatic presentation for Dukes C CRC

- Annual probability of symptomatic presentation for Dukes D CRC
- Annual probability of recurrence after treatment for Dukes D CRC

The BOBYQA optimization achieved a minimized goodness-of fit of 2.27×10^{-6}. As shown in **Table S2**, the model-predicted incidence and mortality risks closely matched the observed targets, with relative errors of +5.2% and −0.6%, respectively, demonstrating a highly robust internal validation.

Table S3. Scenario Analysis: Impact of Varying Screening Initiation Ages on Cost-Effectiveness and Resource Requirements

**Base Case**	**Annual FIT Starting at 40**	**Biennial FIT Starting at 40**
Cost (per person, ¥)	408,058	451,407
QALY (per person)	26.48318394	26.44046574
TCS procedures (per 100,000 persons)	201,836	168,082
**Starting age: 45 years**		
Cost (per person, ¥)	408,555	452,379
QALY (per person)	26.47867546	26.40865321
TCS procedures (per 100,000 persons)	193,136	163,146
vs Annual FIT starting at 40	Dominated	Dominated
vs Biennial FIT starting at 40	Dominant	Dominated
**Starting age: 50 years**		
Cost (per person, ¥)	425,864	465,948
QALY (per person)	26.44234849	26.40894644
TCS procedures (per 100,000 persons)	178,895	152,316
vs Annual FIT starting at 40		Dominated
vs Biennial FIT starting at 40	Dominant	Dominated

Abbreviations: FIT, fecal immunochemical test; QALY, quality-adjusted life-year; TCS, total colonoscopy.

Study Design

This scenario analysis compares costs, QALYs, and TCS requirements for screening initiating at ages 45 and 50 years against the base case (initiation at age 40).

Outcomes and Metrics

- Costs: Represent total cumulative costs per person.
- QALYs: Quality-adjusted life-years gained.
- TCS procedures: Calculated as the total number of procedures per 100,000 population.

Comparison Results

- Dominated: Indicates that the alternative screening yields fewer QALYs at a higher cost compared to the reference screening (Annual or Biennial FIT starting at age 40).
- Dominant: Denotes a screening that yields more QALYs at a lower cost than the reference screening.

REFERENCES

1. Tappenden P, Chilcott J, Eggington S, et al. Option appraisal of population-based colorectal cancer screening programmes in England. *Gut*. 2007;56(5):677-684. https://pubmed.ncbi.nlm.nih.gov/17142648/

2. Sekiguchi M, Igarashi A, Matsuda T, et al. Optimal use of colonoscopy and fecal immunochemical test for population-based colorectal cancer screening: a cost-effectiveness analysis using Japanese data. *Jpn J Clin Oncol*. 2016;46(2):116-215. http://dx.doi.org/10.1093/jjco/hyv186

3. Cancer Statistics. Cancer Information Service, National Cancer Center, Japan (Vital Statistics of Japan, Ministry of Health, Labour and Welfare). Accessed 2025 July 24. https://ganjoho.jp/reg_stat/statistics/data/dl/statistics_p02.html

4. Brenner H, Altenhofen L, Stock C, et al. Natural history of colorectal adenomas: birth cohort analysis among 3.6 million participants of screening colonoscopy. *Cancer Epidemiol Biomarkers Prev*. 2013;22(6):1043-1051. https://pubmed.ncbi.nlm.nih.gov/23632815/

5. Hashiguchi Y, Muro K, Saito Y, et al. Japanese Society for Cancer of the Colon and Rectum (JSCCR) guidelines 2019 for the treatment of colorectal cancer. *Int J Clin Oncol.* 2020;25(1):1-42. http://dx.doi.org/10.1007/s10147-019-01485-z

6. Abridged Life Tables for Japan 2024. Accessed 2026 May 2. https://www.lifetable.de/File/GetDocument/data/JPN/JPN000020242024CU1.pdf

7. Ministry of Health, Labour and Welfare. About the revision of medical fee in FY 2024. Accessed 2025 May 18. https://www.mhlw.go.jp/stf/seisakunitsuite/bunya/0000188411_00045.html

8. Watanabe T, Goto R, Yamamoto Y, et al. First-Year Healthcare Resource Utilization Costs of Five Major Cancers in Japan. Int J Environ Res Public Health. 2021;18(18). http://dx.doi.org/10.3390/ijerph18189447

9. Ministry of Health, Labour and Welfare. Comprehensive Survey of Living Conditions. 2022. Accessed 2025 July 3. https://www.mhlw.go.jp/toukei/saikin/hw/k-tyosa/k-tyosa22/dl/04.pdf

10. Ministry of Health, Labour and Welfare. Report on Regional Public Health Services and Health Promotion Services in 2023. Accessed 2025 May 20. https://www.mhlw.go.jp/toukei/saikin/hw/c-hoken/23/index.html

11. Graser A, Stieber P, Nagel D, et al. Comparison of CT colonography, colonoscopy, sigmoidoscopy and faecal occult blood tests for the detection of advanced adenoma in an average risk population. *Gut*. 2009;58(2):241-248. https://pubmed.ncbi.nlm.nih.gov/18852257/

12. Imperiale TF, Gruber RN, Stump TE, et al. Performance characteristics of fecal immunochemical tests for colorectal cancer and advanced adenomatous polyps: A systematic review and meta-analysis. *Ann Intern Med*. 2019;170(5):319-329. http://annals.org/article.aspx?doi=10.7326/M18-2390

13. Japanese Society of Gastrointestinal Cancer Screening. Daichogan kenshin manyuaru 2021 nendo ban [Manual for colorectal cancer screening 2021 edition] In Japanese] Accessed 2025 Aug 3. https://www.jsgcs.or.jp/files/uploads/d_manualbook2021.pdf

14. Tanaka S, Kashida H, Saito T, et al. Colonic ESD/EMR Guidelines (2nd ed). *Gastroenterol Endosc* 2019;61(6):1321-1344. https://www.jstage.jst.go.jp/article/gee/61/6/61_1321/_html/-char/ja

15. Noto S, Saito S, Shiroiwa T, et al. Estimation of lifetime QALYs based on lifestyle behaviors. *Int J Environ Res Public Health* 2021;18(19):9970. https://pubmed.ncbi.nlm.nih.gov/34639271/

16. Shiroiwa T, Ikeda S, Noto S, et al. Comparison of value set based on DCE and/or TTO data: Scoring for EQ-5D-5L health states in Japan. Value Health. 2016;19(5):648-654. http://dx.doi.org/10.1016/j.jval.2016.03.1834

17. Kameyama H, Shimada Y, Yagi R, et al. Quality of life of patients after colorectal cancer surgery as assessed using EQ-5D-5L scores. *Gan To Kagaku Ryoho*. 2017;44(12):1083-1085. https://mol.medicalonline.jp/library/journal/download?GoodsID=ab8gtkrc/2017/004412/011&name=1083-1085j&UserID=131.113.186.38&base=jamas_pdf

18. Ikeda S, Shiroiwa T, Igarashi A, et al. Developing a Japanese version of the EQ-5D-5L value set. *J Nat Inst Public Health* 2015;64(1):47-55. Japanese. https://www.niph.go.jp/journal/data/64-1/201564010008.pdf
